# Endoscopic ultrasound-guided radiofrequency ablation for pancreatic cystic neoplasms: a comprehensive review

**DOI:** 10.3389/fmed.2026.1865482

**Published:** 2026-06-15

**Authors:** Xinzhu Sun, Nan Ge

**Affiliations:** Department of Gastroenterology, Shengjing Hospital of China Medical University, Shengyang, China

**Keywords:** endoscopic ultrasound-guided radiofrequency ablation, intraductal papillary mucinous neoplasm, mucinous cystic neoplasm, pancreatic cystic lesions, efficacy, safety

## Abstract

The detection of pancreatic cystic lesions (PCLs) is increasingly common, with certain subtypes like intraductal papillary mucinous neoplasms (IPMN) and mucinous cystic neoplasms (MCN) carrying malignant potential. Current management, based on risk-stratified guidelines, faces challenges including imperfect specificity of high-risk features, surgical overtreatment, and the burden of long-term surveillance. For high-risk patients unfit for surgery, the traditional surgery or surveillance paradigm is inadequate, driving the need for minimally invasive therapies. Endoscopic ultrasound-guided radiofrequency ablation (EUS-RFA) merges precise targeting with thermal ablation, emerging as a promising local treatment option for PCLs. This review aims to systematically explore the technical principles, device evolution, and standardized operational procedures of EUS-RFA. It will provide a detailed analysis of its current clinical application status, efficacy, and safety evidence across different pathological types of PCLs, and highlight its unique advantages and applicable scenarios through a comparative analysis with EUS-guided chemical ablation. Finally, this article will examine the current challenges facing this technology and prospect its future development directions. Comprehensive existing evidence suggests that EUS-RFA has evolved from a prospective technology into an important therapeutic option characterized by high efficacy, safety, and minimal invasiveness for carefully selected PCLs with malignant potential. However, the full establishment of its clinical value still relies on the accumulation of higher-level evidence-based data and further standardization in the future.

## 1 Introduction

Pancreatic cystic lesions (PCLs) represent a group of diseases characterized by high heterogeneity in their pathological origin, biological behavior, malignant potential, and clinical outcomes. With the widespread use of cross-sectional imaging and the popularity of health screenings, the incidence of incidentally detected asymptomatic PCLs has risen substantially (1–3). A 2024 proportional meta-analysis encompassing 15 studies and 65,607 subjects revealed that the overall prevalence of PCLs worldwide has reached 13 to 18% (4). With the development of EUS-guided tissue acquisition techniques such as fine-needle aspiration (FNA), the pathological diagnosis of PCLs has also been more standardized (5, 6). PCLs are classified into two categories: non-neoplastic pancreatic cysts and pancreatic cystic neoplasms. The former, which have no malignant potential, include pseudocysts, duplication cysts, retention cysts, and lymphoepithelial cysts. The latter are further subdivided into serous cystic neoplasms (SCN), mucinous cystic neoplasms (MCN), intraductal papillary mucinous neoplasms (IPMN), and solid pseudopapillary neoplasms (SPN), as well as solid tumors with cystic components, such as cystic neuroendocrine tumors (7). It is noteworthy that SPN, while often exhibiting cystic degeneration on imaging, is pathologically distinct as a low-grade malignant solid tumor rather than a typical epithelial cystic neoplasm. Among the diverse classifications of PCLs, IPMN and MCN are the most frequently encountered premalignant lesions in clinical practice (8, 9). At present, the management of PCLs by major international guidelines is primarily based on risk stratification. Surgical resection is recommended for lesions exhibiting “worrisome features” (such as mural nodules, main pancreatic duct dilation between 5–9 mm, cyst diameter ≥3 cm, lymphadenopathy, abrupt change in pancreatic duct caliber with distal pancreatic atrophy) or “high-risk stigmata” (such as obstructive jaundice, enhancing solid components, main pancreatic duct dilation ≥10 mm), as well as for PCLs that have caused related symptoms. In contrast, PCLs with low malignant potential are advised to undergo long-term surveillance (10–12). However, this decision-making framework is facing significant challenges. Firstly, the specificity of imaging and EUS for predicting malignancy is not 100%. This means that some patients undergoing radical pancreatic resection may have their final postoperative pathology confirm benign lesions (13), yet they bear the substantial trauma and perioperative risks. Recent studies indicate that the incidence of severe complications (Clavien-Dindo grade III-V) following pancreaticoduodenectomy ranges from 10 to 25% (14–16). Secondly, imaging surveillance strategy not only imposes a substantial economic burden on the healthcare system (17), but also subjects patients to long-term psychological stress and lifestyle disruptions (18, 19). For elderly patients who cannot tolerate major pancreatic surgery, yet whose lesions carry a clear risk of progression, the traditional decision-making model is no longer applicable. This pressing clinical need has driven the development of minimally invasive interventional therapies for PCLs.

Therapeutic endoscopic ultrasound (EUS) provides an ideal platform for the local treatment of pancreatic diseases by virtue of its ability to visualize the pancreas and surrounding structures with close proximity and high resolution, and to enable precise puncture under real-time guidance (20, 21). Among the various EUS-guided interventional techniques, EUS-guided radiofrequency ablation (EUS-RFA) integrates the superior anatomical localization capability of EUS with the reliable thermal coagulation effect of radiofrequency ablation (RFA). Its fundamental principle involves the precise placement of a radiofrequency electrode into the target component under real-time EUS guidance. The application of a high-frequency alternating current causes ionic friction in the surrounding tissue, generating heat. This leads to irreversible coagulative necrosis of the cells in the target area, thereby achieving the therapeutic goals of inactivating potentially malignant epithelium, eliminating mural nodules, and inducing cyst shrinkage or complete resolution (22). Compared to surgical intervention, EUS-RFA offers the advantages of being minimally invasive, precise, and allowing repeat sessions, while maximizing the preservation of normal exocrine pancreatic function. Since its first description in animal models in 1999 (23), EUS-RFA has undergone a process of device iteration, accumulation of operational experience, and clinical evidence. Its prospects in the treatment of pancreatic lesions, including PCLs, have become increasingly clear (24–27). This review aims to provide a comprehensive overview and summary of the technical basis, clinical evidence, safety, and future prospects of EUS-RFA in the field of PCLs treatment.

## 2 Technical basis of EUS-RFA

### 2.1 Physical and biological principles of RFA

The therapeutic effect of RFA is achieved by converting electrical energy into thermal energy. When a high-frequency alternating current is applied via an electrode, the resistance of the tissue generates heat (Joule heating), leading to cellular damage (28). RFA systems are classified as monopolar, bipolar, or multipolar based on their electrical circuit design, which influences the size, shape, and safety profile of the ablation zone (20, 29). The efficacy of ablation is primarily determined by the achieved tissue temperature. Rapid heating to between 60 °C and 100 °C causes irreversible protein denaturation and coagulative necrosis (30). Research indicates that applying energy at a lower power for a longer duration is often more effective than using high power briefly, as it promotes more uniform thermal conduction and avoids rapid tissue carbonization that can impede energy delivery (31, 32). Beyond this direct thermal effect, RFA may also stimulate a systemic immune response. The necrosis of tumor cells releases antigens and damage signals, which can activate dendritic cells and trigger a tumor-specific T-cell immune response, a phenomenon sometimes described as an “in situ vaccine” effect (33–35).

### 2.2 Endoscopic ultrasound as a guidance platform

EUS guidance provides indispensable advantages for the application of RFA in the pancreas, a retroperitoneal organ adjacent to critical blood vessels and viscera. Primarily, EUS offers high-resolution, real-time imaging of the pancreas and its surrounding structures. This allows the operator to accurately measure cyst dimensions, identify internal structures such as mural nodules and septations, and assess the spatial relationship between the lesion and the pancreatic duct. During the procedure, the color Doppler function can be utilized to clearly identify and avoid adjacent vascular structures (e.g., the splenic artery and vein, superior mesenteric artery and vein) as well as ducts like the main pancreatic duct and common bile duct. This enables precise needle placement and helps prevent inadvertent injury (36, 37). Secondly, by puncturing through the gastric or duodenal wall, EUS-RFA provides a direct, minimally invasive access pathway to pancreas. This is particularly advantageous for reaching surgically challenging regions such as the pancreatic head and uncinate, thereby avoiding the trauma associated with open or laparoscopic surgery (38). Finally, during the ablation procedure, the operator can observe a hyperechoic area that appears due to tissue vaporization or microbubble formation around the electrode on EUS in real time. This allows for the visualization and real-time monitoring of the extent and progression of the ablation (25, 39).

### 2.3 Evolution and comparison of device systems

EUS-RFA devices primarily consist of a high-frequency electrosurgical generator, a puncture guide needle, a radiofrequency ablation catheter electrode, and a dispersive electrode (grounding pad, required only for monopolar probes). Currently, the main types of EUS-guided radiofrequency probes used for the pancreas include the following categories:

#### 2.3.1 Habib™ EUS-RFA catheter(EMcision, London, United Kingdom, recently purchased by Boston Scientific Corp., Marlborough, MA, United States)

This probe is an extremely fine-gauge monopolar probe with a diameter of only 1-Fr and a length between 190 and 220 cm. Its active portion is 20 mm long, and it operates at a high-frequency current of 480 kHz (40). The electrode is typically applied at a power of 5–25 W for 60–120 s, generating a necrotic area of 8–10 mm. The energy is supplied by a universal electrosurgical radiofrequency generator, such as the RITA 1500X or a similar model. Habib™ EUS-RFA does not possess an independent puncturing function. During the procedure, a standard 19G or 22G EUS puncture needle is first used to establish a tract to the lesion. After the stylet is removed, the probe is advanced into the lesion through the needle sheath. Its greatest advantages lie in its excellent compatibility and flexibility, particularly suitable for lesions located in anatomically challenging positions. However, due to its fine diameter and the absence of an active cooling system, its single-application ablation zone is relatively limited. Therefore, operators typically use relatively conservative parameters during the procedure, such as the manufacturer-recommended 10 W power applied for 120 s (41). Multiple ablation applications may be required for large PCLs. It is worth noting that this device was previously withdrawn from the market for modifications.

#### 2.3.2 STARmed EUSRA™ RF system (Taewoong Medical Co., Goyang, South Korea)

This is a radiofrequency ablation needle specifically designed for EUS-guided procedures. It is typically 18G or 19G in gauge and 140 cm in length. The shaft is insulated except for a 5 mm to 30 mm active tip that delivers the current. Its key technical feature is an integrated internal cooling system: during ablation, cooled saline (32 °F) circulates continuously via a peristaltic pump, which lowers the electrode temperature, prevents tissue charring, avoids a rapid rise in impedance, and enables more continuous and effective energy delivery. This results in a larger, more uniform, and controllable ablation zone. The dedicated radiofrequency generator (VIVA generator, 400–500 kHz) monitors tissue impedance and temperature in real time and automatically adjusts or stops energy output if safety thresholds are exceeded (impedance ≥ 500 Ω; temperature > 100 °C), thereby improving efficiency, safety, and convenience while reducing risks associated with excessive tissue vaporization.

The two devices mentioned above are currently the more commonly used ones in research and are also the only two devices approved by the U. S. FDA. They are both monopolar probes. During operation, a grounding pad must be attached to the body surface to form a complete electrical circuit. Other devices include the 19-gauge EUS-FNA needle electrode (Radionics, Inc., Burlington, MA, United States) and the Hybrid-cryotherm probe (ERBE Elektromedizin GmbH, Tübingen, Germany) (42). The latter is a bipolar probe, where the current flows between its two electrodes, eliminating the need for a grounding pad. Theoretically, this design carries a lower risk of thermal damage to surrounding non-target tissues (43). Its generator (VIO 300D, ERBE Elektromedizin GmbH, Tübingen, Germany) integrates a carbon dioxide cooling system (44). Future devices may integrate real-time temperature and resistance monitoring, multi-polar radiofrequency probes, and even artificial intelligence-assisted ablation boundary prediction systems (45), in pursuit of superior ablation efficacy, enhanced controllability, and improved maneuverability.

### 2.4 Procedural workflow and technical considerations

#### 2.4.1 Preoperative assessment and preparation

##### 2.4.1.1 Preoperative diagnosis

Accurate preoperative diagnosis is essential for selecting appropriate EUS-RFA candidates, as misdiagnosis of pancreatic cysts remains common. Surgical series indicate a preoperative misdiagnosis rate of approximately 19%, leading to significant clinical management errors (46). EUS-guided cyst fluid analysis provides crucial adjunctive diagnostic information. Intra-cystic glucose level has proven to be a highly discriminant biomarker for identifying mucinous lesions (47). Utilizing such objective biomarkers is crucial to ensure that EUS-RFA is targeted at lesions with confirmed malignant potential.

##### 2.4.1.2 Patient selection

There are currently no unified standards for indications and contraindications for EUS-RFA in the treatment of PCLs. The criteria, based on clinical experience and literature review (41, 42, 48), are summarized in Table 1.

**Table 1:** Indications and contraindications for EUS-RFA in the treatment of PCLs.

Indications	Contraindications
PCLs with clear malignant potential	Uncorrectable coagulopathy (PT INR >1.5 and platelet count <50,000/μL)
Inability to tolerate or refusal of surgical resection	Unability to discontinue anticoagulant/antiplatelet medications preoperatively
A safety margin of >2 mm between the lesion and the main pancreatic duct	Patient in poor general condition, unfit for the procedure
	Presence of an implanted cardiac pacemaker
	Direct communication between the cystic lesion and the main pancreatic duct
	Inability to obtain a safe puncture tract
	Failure to provide signed informed consent

PCLs pancreatic cystic lesions.

##### 2.4.1.3 MDT discussion

A multidisciplinary team (MDT) comprising gastroenterology, pancreatic surgery, radiology, and pathology experts should review the case to confirm the treatment rationale, evaluate lesion characteristics, and weigh the anticipated benefits against the potential risks of EUS-RFA.

##### 2.4.1.4 Patient preparation

Standard preoperative preparation includes obtaining detailed informed consent, enforcing a 6–8 h fast, and administering prophylactic intravenous antibiotics (e.g., a quinolone) along with a rectal non-steroidal anti-inflammatory drug (NSAID, e.g., indomethacin) to mitigate the risks of procedure-related infection and pancreatitis (49, 50).

##### 2.4.1.5 Equipment preparation

All necessary equipment, including the therapeutic echoendoscope, radiofrequency generator, the selected EUS-RFA probe, and any ancillary devices (e.g., grounding pad, peristaltic pump), must be confirmed to be functional and ready for use prior to the procedure.

#### 2.4.2 Intraoperative procedure

##### 2.4.2.1 Lesion re-evaluation and path planning

The patient is placed in the left lateral decubitus position. If a monopolar RFA needle is to be used, the entire surface of the grounding pad should be firmly attached to the patient’s body, preferably placed as close as possible to the operative field (e.g., the lower back), to prevent skin burns. Under sedation or general anesthesia, a detailed EUS examination is first performed to precisely measure the three-dimensional dimensions of the cyst and to confirm the internal structures, including septations, mural nodules, calcifications, and vascularity. The Doppler mode is used to scan the surrounding vessels and the course of the pancreatic duct, in order to select the shortest, straightest puncture tract that avoids critical structures.

##### 2.4.2.2 Cyst fluid aspiration

A 19G or 22G EUS-FNA needle is first used to puncture the center of the cyst cavity via a transgastric or transduodenal approach under real-time guidance. Cyst fluid is aspirated until only a small amount remains for subsequent RFA targeting. This step serves a dual purpose. Firstly, the aspirated fluid is sent for biochemical analysis (carcinoembryonic antigen/CEA, glucose, amylase), cytology, and genetic testing, providing critical diagnostic information (51–55). Secondly, it creates space for the subsequent ablation, reduces the heat sink effect caused by the fluid, thereby ensuring that thermal energy is applied fully and evenly to the cystic wall epithelium and improving ablation efficacy. For multilocular PCLs, the needle tip must be moved in a fanning motion to fenestrate the septa and achieve adequate drainage (56).

##### 2.4.2.3 RFA probe placement and energy application

For needle-type EUS-RFA electrodes, the EUS-FNA needle is withdrawn and replaced with the RFA probe. The active tip length is selected based on lesion size. Following Doppler confirmation of no intervening vessels, the probe is advanced into the cyst along the original or a slightly adjusted tract. The active portion must be optimally positioned within the cyst (e.g., targeting a mural nodule or centered in the cavity) and must not extend beyond the lesion. If it does, the needle is retracted under EUS guidance until the tip is within the lesion margins. For the catheter-based Habib™ EUS RFA electrode, the procedure differs: the stylet is removed from the pre-positioned EUS-FNA needle. The electrode catheter is then advanced through its lumen until it can go no further. While the Habib catheter is held steady, the outer FNA needle is withdrawn to expose the active tip. Fluoroscopic guidance may assist in visualizing the probe tip as it protrudes from the needle (57).

##### 2.4.2.4 Parameter setting and energy application

Parameters for the radiofrequency generator are set based on the type of probe used, the size, and the morphology of the cyst to deliver high-frequency current energy for ablation. For EUSRA™ RF system, a common power setting is 50 W, and the application time is variable, typically ranging between 10 and 120 s. For Habib™ EUS-RFA, a lower power is commonly used, such as 10 W, applied for 120 s. The size of the ablation zone depends on the generator power, the RFA needle tip length, and the duration of energy application. A safety margin of 5 mm from adjacent structures is recommended (42). Studies have shown that a thermal injury that penetrates deeper but spreads more slowly can be achieved by using lower power and applying it for a longer duration, making the procedure safer (32). After the delivery of radiofrequency energy is completed, it is recommended to wait for a few seconds before retracting the device to avoid thermal injury and potential perforation of the gastrointestinal wall traversed by the needle (41).

##### 2.4.2.5 Real-time monitoring and multiple applications

Once ablation begins, EUS imaging shows the tissue around the electrode tip changing from hypoechoic to hyperechoic, confirming effective treatment. The procedure must be stopped if vigorous hyperechoic bubbling is seen or if impedance exceeds 500-1000 Ω (58). For small PCLs (<1 cm), the needle tip can be centered. For larger cysts (>2 cm), multiple ablations are typically needed to cover the entire cyst wall by repositioning the needle or adjusting its angle. Both the long axis and perpendicular axis approaches can be utilized for precise probe placement and lesion targeting. The long axis approach involves using the endoscopic elevator and the large and small dials to position the probe along the longitudinal axis of the cyst, achieving linear alignment and effective energy delivery. It is recommended to begin the ablation at the distal-most aspect of the lesion, then gradually withdraw the needle, as the gas artifact generated by the initial ablation may obscure visualization of deeper structures. Additional punctures can be performed as needed to achieve complete treatment. When utilizing the perpendicular axis approach, it is necessary to rotate the endoscope shaft or adjust the operator’s body axis to orient the probe perpendicularly to the long axis of the cyst (59). Following RFA, contrast-enhanced harmonic EUS (CEH-EUS) can assess the ablation zone in real time to detect residual viable tissue and guide the need for further treatment (60). However, some studies recommend that CEH-EUS should be performed at least 1 week after the procedure, as acute post-RFA hyperemia may otherwise interfere with image interpretation (61). At the centers where CEH-EUS is not available, contrast-enhanced CT can be performed to assess treatment efficacy.

#### 2.4.3 Post-procedure management

**Figure 1:**
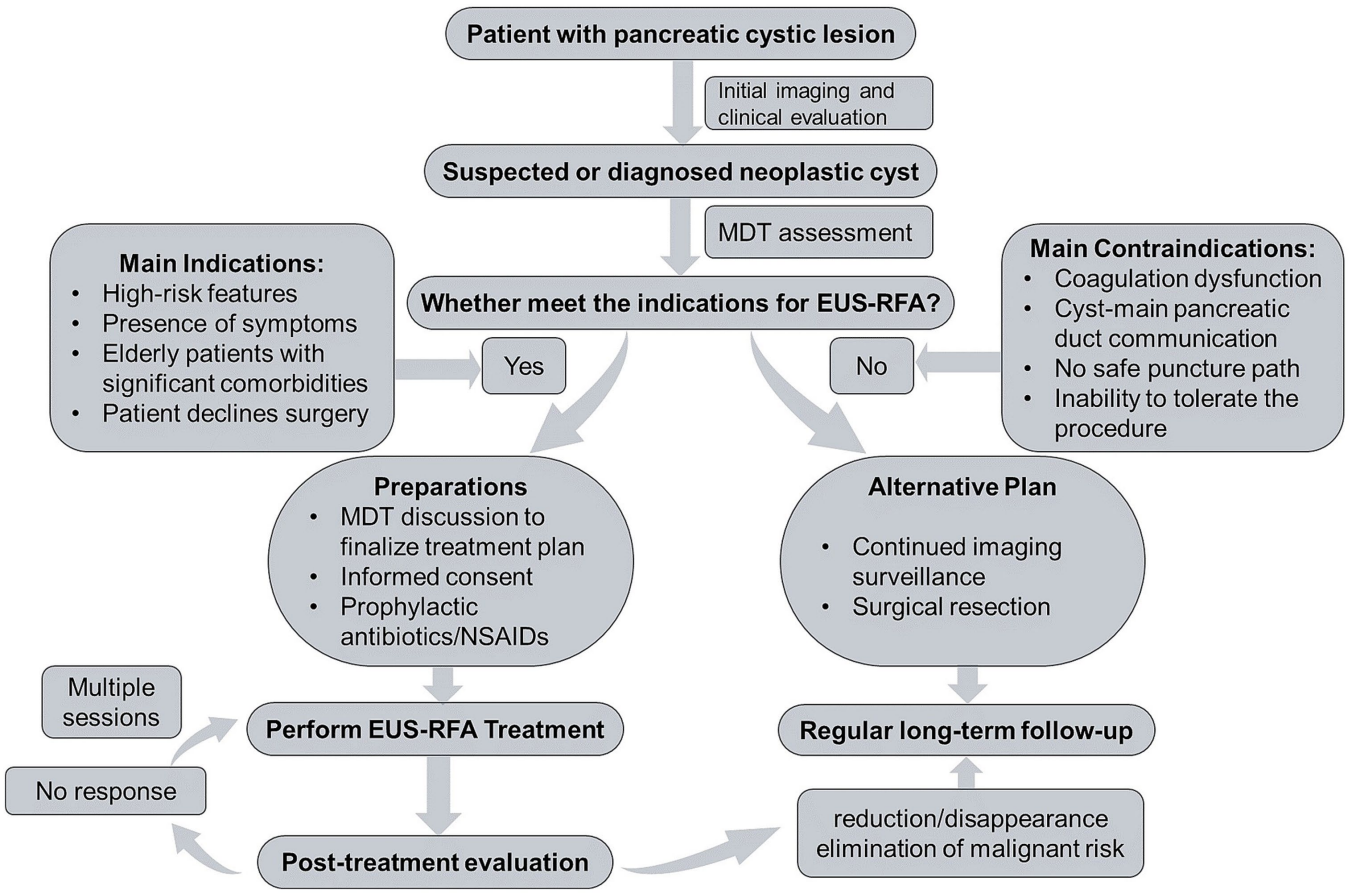
Evaluation and management algorithm for EUS-RFA of PCLs.

After the procedure, patients require hospitalization for observation, with monitoring of vital signs and symptoms (such as abdominal pain, fever, nausea, and vomiting). Routine blood tests for serum amylase and lipase are performed 4–6 h post-procedure and on the following morning to rule out asymptomatic hyperamylasemia or acute pancreatitis. Patients experiencing persistent or severe abdominal pain should promptly undergo abdominal CT to exclude serious complications such as perforation or bleeding. Following discharge, patients are scheduled for follow-up at 1 month, 4–6 months, and annually thereafter. Cross-sectional imaging and/or EUS is performed to confirm complete ablation and assess for recurrence. Repeat ablation may be performed if necessary (42). An evaluation and management algorithm for EUS-RFA of PCLs is shown in Figure 1.

## 3 Clinical applications and efficacy evidence of EUS-RFA in different types of pancreatic cystic lesions

### 3.1 Premalignant lesions: IPMN and MCN

IPMN and MCN are the primary pancreatic cystic neoplasms targeted by EUS-RFA due to their established malignant potential (62–64). IPMN is classified by involvement site into main duct type (MD-IPMN), branch duct type (BD-IPMN), and mixed type (mix-IPMN), with MD-IPMN and mix-IPMN carrying the highest risk of malignancy (62, 65). For MCN, the absence of communication with the main pancreatic duct and the presence of ovarian-type stroma are defining features, and its size (>4 cm) or the presence of mural nodules/solid components significantly increases malignant risk (63, 64, 66, 67). The treatment objective of EUS-RFA for IPMN and MCN is to eliminate or ablate the mucinous epithelium with high-grade dysplasia within the cyst wall through the thermal coagulation effect, particularly targeting mural nodules associated with malignant risk. The ultimate goal is to eradicate their malignant potential, rather than the mere pursuit of complete cyst disappearance on imaging.

The landmark first multicenter prospective study, published by Pai et al. in 2015, holds significant importance (57). This study enrolled six patients with PCLs who were unsuitable for surgery (four with MCN, one with IPMN, and one with microcystic adenoma). Using a 19G or 22G FNA needle to place the Habib™ EUS-RFA catheter, a single session of radiofrequency ablation was performed on each PCL. The generator power was set between 5 and 25 W. Follow-up at 3–6 months post-procedure showed that two patients (33%) achieved complete remission (CR), while three patients achieved partial remission (PR) with an average cyst size reduction of 48.4%. No serious adverse events such as post-procedure pancreatitis, perforation, or bleeding occurred within 48 h after the procedure. Only two patients experienced mild, self-limiting abdominal pain. This study preliminarily demonstrated the feasibility of applying EUS-RFA to PCLs and its good short-term tolerability. However, the study did not provide long-term follow-up data to evaluate recurrence.

In 2019, a prospective, multicenter, open-label study by Barthet et al. included 17 patients with PCLs (16 IPMNs, one mucinous cystadenoma) (49). Ablation was performed with an 18G STARmed EUSRA™ RF probe at 50 W, maintaining a 2 mm safety margin. At six-month follow-up, eight patients (47.1%) achieved complete response (CR), three (17.6%) had partial response (PR, >50% cyst reduction), and six (35.3%) experienced treatment failure. By 12 months, the CR rate rose to 65% (11/17), and the overall significant response rate (CR + PR) reached 71% (12/17), suggesting a delayed response possibly related to post-ablation immune activation. Notably, mural nodules were completely eliminated in all 12 baseline cases (100% ablation rate), underscoring the technique’s potential to mitigate malignant risk. Adverse events included one case of pancreatitis with fever and one pneumoperitoneum with fluid collection. Subsequently, the protocol was amended to include pre-procedural rectal diclofenac, intravenous antibiotics, and cyst fluid aspiration, after which no further adverse events occurred. Long-term follow-up (median 42.6 months) published in 2021 confirmed durability. Among 15 evaluable patients, 40% (6/15) maintained CR and 26.6% (4/15) maintained PR, with no mural nodule recurrence (56). The systemic immune response triggered by RFA may be part of the reason for the long-term efficacy.

The study by Younis included a total of five patients with PCNs (four with IPMNs, one with MCN; three of the IPMNs had mural nodules) (68). RFA was performed using a 19G STARmed EUSRA™ RF system with a 10-mm actively cooled tip in continuous mode at 50 W. Complete radiographic resolution was achieved in 60% of patients, with 20% partial response and 20% treatment failure. The adverse event rate was 60% (two cases of abdominal pain, one pancreatitis), all mild and managed conservatively. In the observational retrospective study by Napoleon et al., 10 cases of IPMN with mural nodules and one case of SPN undergoing EUS-RFA were included. Among the 10 IPMN patients, two were lost to follow-up. Among the remaining patients, the complete response rate was 62.5% (5/8), the partial response rate was 37.5% (3/8), and the mural nodule elimination rate reached 100%, indicating effective ablation (69). The study by De La included a total of six CMNs located in the head, body, or uncinate process of the pancreas (median size 21.5 mm, range 13–28 mm). One case of complete response and five cases of partial response were observed. Response criteria were defined as follows: A complete response was defined as the absence of an enhancing area on imaging and no evidence of residual malignant tissue on biopsy. A partial response was defined as a reduction in diameter of 50% or more (70).

A 2026 study specifically investigated EUS-RFA for the treatment of large (≥4 cm, mean 4.6 cm), predominantly multilocular (80%) BD-IPMNs (59). Using the VIVA combo system with a 19G STARmed EUSRA™ RF 10 mm electrode, RFA was delivered at 50 W in continuous mode, limited to 45 s or an impedance of 400 Ω. Efficacy was assessed from two aspects: computerized 3D volumetric analysis based on follow-up MRI or CT scans and cyst-fluid next-generation sequencing (NGS) evaluation for KRAS and GNAS mutations. Over a mean 18-month follow-up, 78.6% (22/28) of lesions achieved a partial volumetric response (≥50% reduction), including 39.3% (11/28) with a complete response (≥90% reduction). On molecular analysis, 88.2% of cases showed >90% reduction in mutation VAF, and all three baseline high-risk mutations became undetectable post-treatment. Adverse events occurred in 12.2% of sessions (5/41) and 20% of participants (5/25), all managed non-surgically. These events were predominantly technical complications related to the RFA probe, including insulation damage, needle bending, and one case of severe acute pancreatitis with duodenal perforation. Furthermore, technical optimization was also explored. Excessive use of the elevator during EUS-RFA procedures may lead to probe malfunction. Therefore, in subsequent operations, the researchers minimized elevator usage and adopted alternative maneuvers such as torquing the endoscope shaft or partial withdrawal of the endoscope. This effectively reduced the risk of probe damage while maintaining precise lesion targeting.

### 3.2 Benign symptomatic lesions: SCN

SCN is a benign pancreatic cystic neoplasm composed of glycogen-rich epithelial cells, and its cysts are filled with serous fluid. Based on cyst morphology, SCNs are primarily classified into three types: microcystic, macrocystic (oligocystic), and solid. SCNs are typically asymptomatic, are more common in middle-aged and elderly women, and carry an extremely low risk of malignant transformation, usually warranting only periodic surveillance. However, intervention is indicated when SCNs become very large (>4 cm) and cause significant compressive symptoms (such as abdominal pain, early satiety, or obstructive jaundice). For symptomatic SCN patients who are unwilling or unsuitable for surgery, EUS-RFA can serve as an effective minimally invasive option for palliative treatment.

In a 2021 retrospective study by Oh et al., EUS-RFA was used to treat 13 cases of microcystic SCN presenting with a typical honeycomb morphology (71). The results showed that, after a mean follow-up of 9.2 months, although no patient achieved complete radiological response, the median cyst volume measured on CT images significantly decreased from 37.82 mL to 10.95 mL, with a partial response rate of 61.5% (8/13). More importantly, all patients experienced significant relief of compressive symptoms following the reduction in cyst volume. The researchers analyzed that the numerous fibrous septa within microcystic SCNs might limit effective heat conduction between different cystic cavities, thereby making complete ablation challenging. However, this does not diminish its effectiveness as a palliative debulking therapy. Regarding adverse events, only one case of postoperative self-limiting abdominal pain was reported.

### 3.3 Low-grade malignant lesions: SPN

SPN is a rare pancreatic tumor of low malignant potential, predominantly occurring in young women, and typically presents as a solitary mass in the body or tail of the pancreas. Morphologically, SPNs are classified into solid, pseudopapillary, and mixed types (72). Although surgical resection remains the gold standard treatment for SPN, EUS-RFA is emerging as a promising minimally invasive therapeutic option for small (≤2 cm) lesions or patients ineligible for surgery (73). Choi et al. (74) reported two cases of SPN treated with EUS-RFA after the patients declined surgery. Both procedures were completed successfully without any procedure-related adverse events. After a median follow-up of 13 months, one patient (50%) achieved complete radiologic response, while the other showed no response. Another case series demonstrated that imaging studies revealed no residual lesions in three patients with SPNs at the pancreatic head (11–20 mm) during 1–2 years of follow-up after EUS-RFA treatment (75).

### 3.4 Critical appraisal of clinical evidence and limitations

While existing studies confirm the feasibility, efficacy, and acceptable safety of EUS-RFA for PCLs, the current evidence base has significant limitations and heterogeneity that affect the generalizability and certainty of conclusions. The majority of evidence derives from single-center, small-sample prospective studies, retrospective analyses, and case reports, lacking large, multicenter randomized controlled trials (RCTs) directly comparing EUS-RFA with surgical resection or active surveillance. This structure introduces selection bias and center-specific expertise variations. Enrolled patients are often elderly and at high surgical risk, whose cyst biology may not represent the broader patient population.

Definitions of “treatment response” vary across studies (e.g., complete resolution based on radiologic disappearance, percentage volume reduction, or combined molecular clearance), as do follow-up intervals (3–12 months) and assessment modalities (CT, MRI, EUS). This complicates cross-study comparison and meta-analysis. For pre-malignant lesions like IPMN and MCN, the ultimate goal is long-term cancer prevention. Current studies using radiologic response and short-term (1–4 years) progression-free survival as primary endpoints cannot equate to long-term oncologic success. Pancreatic cancer progression often spans decades; thus, the long-term (≥5–10 years) oncologic outcomes of EUS-RFA require extended follow-up data for confirmation.

The role of EUS-RFA as an alternative for surgery-eligible PCLs remains undefined. Surgery offers definitive pathology and theoretically the highest local control but carries significant morbidity. Active surveillance avoids immediate risks but entails anxiety, long-term burden, and progression risk. EUS-RFA aims to balance these, but its risks of incomplete ablation, inability to address occult invasive cancer, and efficacy for multifocal disease are core controversies versus surgery. Future RCTs based on precise risk stratification are needed to directly compare EUS-RFA, surgery, and surveillance in specific subgroups (e.g., BD-IPMN with a single “worrisome feature”) regarding cost-effectiveness and patient-reported outcomes.

## 4 Safety analysis and prevention/management of adverse events

### 4.1 Incidence of adverse events

The safety of EUS-RFA is a paramount consideration. Adverse events associated with EUS-RFA arise from the thermal effects on pancreatic/peripancreatic tissues and the procedure itself (58). Pooled data from available studies indicate an overall adverse event rate ranging from 14 to 22%, with the vast majority being mild to moderate in severity. Severe adverse events are rare, occurring in only 0.5 to 0.9% of procedures. The most common adverse event is mild, self-limiting abdominal pain, followed by acute pancreatitis, which has a reported incidence of 7.9 to 10% (42). Other reported complications include gastric and intestinal wall burns, bleeding, peritonitis, pancreatic duct stenosis, duodenal stricture, and infection. Procedure-related mortality is exceedingly rare, with a single reported death in a 97-year-old patient with multiple comorbidities (76).

A recent meta-analysis pooled data from 31 PCLs across four studies (49, 57, 70, 74) demonstrating a weighted pooled response rate of 76.8%. While the adverse event rate for pancreatic cystic neoplasms was not reported separately, it indicated that the overall complication rate of EUS-RFA applied to pancreatic lesions is relatively low, at approximately 6.7% (77). A recent review summarized data from 46 PCLs treated with EUS-RFA, reporting an adverse event rate as high as 23.2%. However, only 2.1% of these were classified as AGREE grade III/IV events (36), demonstrating a low probability of severe adverse events.

### 4.2 Predictors of adverse events

The most consistently identified and significant predictor is the proximity of the lesion to the main pancreatic duct (MPD). A national French study (RAFPAN) identified a distance of ≤1 mm from the MPD as an independent risk factor for adverse events (OR 4.10) (69). Similarly, other analyses have found that 89% of patients who developed post-RFA pancreatitis had lesions located <2 mm from the MPD (48). For cystic lesions, failure to aspirate the cystic fluid component before ablation has been associated with severe complications like infected necrosis, likely due to the heat-sink effect and increased risk of infection. Specific cyst characteristics may also influence risk; for instance, the presence of enhancing mural nodules, thick walls, thick septations, MPD stricture with pancreatic tail atrophy, and significant solid components within the cyst are considered relative contraindications and may portend a higher risk of complications (78).

### 4.3 Preventive strategies and management

Routine preventive measures include preoperative antibiotics, rectal indomethacin, and cyst fluid aspiration prior to ablation. Procedural optimizations, such as reducing the RFA electrode tip temperature, using devices equipped with internal cooling systems, and maintaining a safe distance from peripancreatic vessels, can further lower the risk of complications (36, 79). For lesions adjacent to the main pancreatic duct, a careful risk–benefit assessment is needed. Prophylactic pancreatic duct stenting may lower pancreatitis risk but must be balanced against the stent-related pancreatitis risk itself. Management of adverse events is based on severity: mild abdominal pain or pancreatitis is treated conservatively (fasting, intravenous fluid resuscitation, analgesia). Active bleeding can be controlled with endoscopic epinephrine injection or hemoclips. Infected or symptomatic peripancreatic fluid collections may be drained under EUS guidance or percutaneously. Pancreatic/biliary duct obstruction warrants endoscopic retrograde cholangiopancreatography (ERCP) with stent placement. Surgery is reserved for severe cases such as intestinal perforation.

Overall, EUS-RFA provides a relatively effective and safe minimally invasive therapeutic modality for PCLs, particularly for patients who are unsuitable for or decline surgical resection. The detailed data from published studies on the application of EUS-RFA in PCLs are summarized in Table 2. Furthermore, multiple clinical trials (such as NCT03417843, NCT02330497, NCT05961982) are currently evaluating the safety and efficacy of EUS-RFA for the treatment of PCLs (80). In the future, more high-quality prospective multicenter clinical studies are expected to provide evidence supporting the application of this technology in PCLs.

**Table 2:** Tabulated summary of clinical outcomes from studies on EUS-RFA for PCLs.

Reference (Author, Year)	Study design	No. of patients	No. of lesions (Procecures)	Types of lesions (n)	Mean Size ± SD (Range) in mm	Location (n)	Device	Complete response	Partial response	Adverse events	Follow-up in months
Krishna et al. (59)	Prospective, single-center	25	30 (41)	BD-IPMN	46 ± 17	Head/Uncinate (21) Body/Tail (9)	STARmed EUSRA™ RF system (50 W)	39.3% (11/28) Missing data: 2	78.6% ^a^ (22/28) Missing data: 2	12.2% (5/41)	18
Polkowski et al. (85)	Case report	1	1 (1)	SPN (1)	13	Head	STARmed EUSRA™ RF system (50 W) + Ethanol ablation after 6 months	100% (1/1)	0% (0/1)	0% (0/1)	12
Napoléon et al. (69)	Retrospective, multicenter	11	11	IPMN (10) SPN (1)	IPMN: 29 SPT: 9	NR	STARmed EUSRA™ RF system (50 W)	62.5% (5/10) Missing data: 1	37.5% (3/10) Missing data: 1	NR	13
Coupier et al. (75)	Case series	3	3 (4)	SPN (3)	16.7 (11–20)	Head (3)	STARmed EUSRA™ RF system	100% (3/3)	0% (0/3)	0% (0/4)	12–24
Younis et al. (68)	Prospective, single-center	5	5 (8)	IPMN (4) MCN (1)	36 (12–60)	Head (3) Body (2)	STARmed EUSRA™ RF system (50 W)	60% (3/5)	20% (1/5)	60% (3/5)	EUS: 7 (4–19), imaging:13 (3–19)
Oh et al. (71)	Retrospective, single-center	13	13 (19)	SCN (13)	50 (34–52.5)	Head (5) Body/Tail (8)	STARmed EUSRA™ RF system	0% (0/13)	61.5% (8/13)	5.3% (1/19)	9.2
Barthet et al. and (49) Barthet et al. (56)	Prospective, multicenter	17	17 (17)	IPMN (16) MCN (1)	28 (9–60)	Head (10) Body (4) Tail (3)	STARmed EUSRA™ RF system (50 W)	40% (6/15) Missing data: 2	26.7% (4/15) Missing data: 2	5.9% (1/17)	42.6
Choi et al. (74)	Prospective, single-center	2	2 (2)	SPN (2)	21.5 (20–23)	Head (1) Tail (1)	STARmed EUSRA™ RF system (50 W)	50% (1/2)	0% (0/2)	0% (0/2)	13
De la Serna et al. (70)	Prospective, single-center	6	6 (8)	MCN (6)	20.7 (13–28)	Head (2) Body (3) Uncinate (1)	STARmed EUSRA™ RF system (50 W)	16.7% (1/6)	83.3% (5/6)	0% (0/6)	8.7
Feng et al. (84)	Case report	1	1 (1)	SCN (1)	35	Tail	Habib™ EUS RFA + lauromacrogol ablation after 2 months	100% (1/1)	0% (0/1)	0% (0/1)	3
Thosani et al. (90)	Retrospective, multicenter	1	1	SCN (1)	23	NR	Habib™ EUS RFA	NR	NR	NR	5
Goyal et al. (91)	Case series	2	2	MCN (2)	20.5	Body (1) Tail (1)	Habib™ EUS RFA (10 W)	NR	NR	NR	NR
Pai et al. (57)	Prospective, multicenter	6	6	IPMN (1) MCN (4) Microcystic SCN (1)	36.5 ± 17.9	NR	Habib™ EUS RFA (5–25 W)	33.3% (2/6)	50% (3/6)	33.3% (2/6)	3–6

^a^In this study, partial response was defined as ≥50% volume reduction, which included lesions that also met the criteria for complete response (≥90% reduction). In other studies presented in this table, partial response typically denotes a response that meets a certain threshold but does not fulfill the criteria for complete response. SD standard deviation; BD-IPMN branch-duct intraductal papillary mucinous neoplasm; IPMN intraductal papillary mucinous neoplasm; MCN mucinous cystic neoplasm; SCN serous cystic neoplasm; SPN solid pseudopapillary neoplasm; NR not reported.

## 5 Comparative perspective with EUS-guided chemical ablation

EUS-guided chemical ablation is another important minimally invasive method for treating PCLs. This technique involves the real-time EUS-guided injection of chemical ablative agents into the cyst cavity to destroy the cyst wall lining epithelium. Commonly used ablative agents include absolute ethanol, paclitaxel, and lauromacrogol (81). Compared to EUS-RFA, EUS-guided chemical ablation was applied to PCLs earlier, and there are more related clinical studies and reports. A recent review summarized data from a total of 347 PCL patients across these studies, including those with MCNs, IPMNs, SCNs, pseudocysts, and cysts of other uncertain nature. The results showed that EUS-guided chemical ablation resulted in complete resolution of the cyst in 63.6% of cases (82).

The key distinctions between EUS-RFA and EUS-guided chemical ablation in terms of mechanism, suitability for different lesion types, and treatment outcomes are summarized in Table 3. A 2023 meta-analysis encompassing 15 studies reported that at a follow-up of ≥12 months, PCLs treated with EUS-guided ablation showed a complete resolution rate of 44%, and a partial response rate (volume reduction of ≥50%) of 30%. Subgroup analysis revealed that the resolution rate for RFA alone (13%) was significantly lower than that for ethanol ablation (32%) and ethanol-paclitaxel combination ablation (70%) (83). This is primarily attributed to the fluidity of the chemical ablation agents, which allows them to contact a larger area of the cyst wall lining, producing sustained chemical necrosis, especially when combined with the cytotoxic drug paclitaxel. However, EUS-RFA possesses an unparalleled advantage in eliminating solid components such as mural nodules (with a nearly 100% efficacy rate), which are difficult for chemical agents to penetrate and inactivate.

**Table 3:** Comparison between EUS-RFA and EUS-guided chemical ablation for PCLs.

Dimension	EUS-RFA	EUS-guided chemical ablation
Mechanism	Thermal effect (coagulative necrosis).	Chemical effect (epithelial necrosis and fibrosis).
Agents	Not applicable (electrical energy).	Absolute ethanol, paclitaxel, lauromacrogol, etc.
Technical features	Requires dedicated RF generator and electrode; parameters adjustable; some with in ternal cooling to enhance safety.	Technically simpler; lacks unified standards for dose, concentration and dwell time.
Indications	IPMNs and MCNs containing mural nodules, solid-predominant SPNs, and difficult locations.	Purely cystic, non-solid, regularly shaped MCNs or larger SCNs.
Coverage of cyst wall	Coverage depends on precise electrode placement. Irregular or multilocular cysts may requires multiple needle insertions.	Potential advantage. Fluid agents can contact a larger area of the cyst wall, offering more comprehensive epithelial coverage.
Efficacy on solid components	Highly effective (near 100% eradication of mural nodules).	Limited efficacy (poor penetration into solid nodules).
Resolution rate	A meta-analysis shows a lower complete resolution rate (13%) of EUS-RFA compared to ethanol ablation (32%) and ethanol-paclitaxel combination (70%) (83). However, prospective studies focusing on IPMNs and MCNs report higher rates of 40–65% at mid-term follow-up with a delayed response (56).	
Potential for combined therapy	Sequential use: RFA first for septa or mural nodules, then chemical ablation for residual epithelium. Small-sample success reported (84, 85).	

For pancreatic tumors with complex structures or where a single ablation session might be incomplete, a combined strategy employing EUS-RFA and chemical ablation is technically feasible. A 2018 case report first described the combined use of EUS-RFA and lauromacrogol ablation for a SCN at the pancreatic tail. The patient initially underwent RFA to address intracystic septations, followed by supplementary lauromacrogol injection 2 months later. A three-month follow-up MRI showed complete resolution of the lesion, with no adverse events reported (84). A 2026 case report describes a sequential therapy used to treat a 13 mm solid pseudopapillary neoplasm in the pancreatic head. An 8 × 7 mm residual lesion was detected 6 months after the initial EUS-RFA. The patient subsequently underwent EUS-guided ethanol ablation, ultimately achieving complete resolution 12 months after the first treatment. The entire process was completed without complication (85). Combined ablation may exert a synergistic effect: RFA can effectively treat the solid components or septations of the lesion, while subsequent chemical ablation can target any residual active epithelial cells or areas that are difficult for RFA to cover uniformly, thereby improving the complete ablation rate. However, all this evidence comes from small-sample case reports, and the timing, sequence, ablation parameters, and long-term efficacy of the combined therapy have not yet been standardized.

## 6 Current limitations, challenges, and future prospects

A core challenge in EUS-RFA for PCLs is the absence of a globally standardized treatment protocol. International consensus is lacking on critical ablation parameters, such as the optimal radiofrequency power, the duration of energy application, and the endpoint criteria for assessing ablation adequacy. Current clinical practice of EUS-RFA is heavily reliant on the individual experience, the conventions of medical centers, and the recommended settings from the particular device manufacturer. This has directly led to significant heterogeneity in data across different studies, making results difficult to compare directly, and has also created obstacles for the standardized training and widespread adoption. A more fundamental limitation is that the long-term oncological efficacy of EUS-RFA still requires evidence from longer-term studies for final confirmation. For premalignant lesions such as IPMN and MCN, the gold standard for evaluating the success of the reduction in future risk of invasive pancreatic cancer. While mid-term data [e.g., Barthet et al. (56)] are encouraging, the progression of pancreatic cancer is slow, evolving over a decade or longer. Therefore, to establish the role of EUS-RFA in cancer prevention and its potential to replace prophylactic surgery in selected patients, large-scale prospective studies with follow-up exceeding five, and preferably 10, years are imperative.

Refining patient selection remains a significant challenge in clinical decision-making. Future efforts should focus on constructing a predictive model that integrates multi-dimensional data: (1) detailed radiomic features (e.g., mural nodules, cyst-main pancreatic duct communication, cyst wall thickness and enhancement patterns, overall morphological complexity); (2) liquid biopsy biomarkers from cyst fluid, including CEA levels and genetic mutations (e.g., KRAS/GNAS), for objective malignant risk stratification; (3) key anatomical factors (lesion location in head/tail, precise distance from main pancreatic duct and major vessels) to assess technical difficulty and weigh benefits against risks; and (4) individual patient factors (age, comorbidities, clinical status, and treatment preferences). Such a multi-omics, multimodal approach is essential to advance from population-based guidelines to individualized precision therapy. Furthermore, rigorous health economics evaluations are still lacking. The long-term cost-effectiveness of EUS-RFA compared to surgery or surveillance requires confirmation through detailed model-based analyses to inform healthcare payers and policymakers.

Future progress in this field hinges on high-level clinical research. Priority should be given to well-designed, large-scale, multicenter, prospective randomized controlled trials comparing long-term cancer incidence and overall survival between EUS-RFA and surveillance in high-risk PCL patients not requiring immediate surgery, or comparing EUS-RFA versus surgical resection in elderly, high-risk patients. Grade I evidence from such trials will determine whether EUS-RFA is included in international guidelines as a standard option. Technological and device innovation will also help overcome current limitations. Closed-loop ablation systems integrating real-time multi-point temperature monitoring and impedance feedback could enable automatic power adjustment and precise ablation control. Fusion navigation platforms incorporating contrast-enhanced ultrasound or elastography may allow intraoperative visualization of ablation boundaries and perfusion changes for real-time efficacy assessment. New probe designs, such as electrodes with adjustable angles and shapes, could better adapt to irregular, multilocular complex PCLs.

Meanwhile, exploring innovative combination therapy strategies may open new pathways. For instance, after the main thermal ablation with EUS-RFA is completed, the cyst cavity could be perfused with sustained-release chemotherapeutic agents (such as nanoparticle-encapsulated gemcitabine) or immunomodulators. Sequential or synergistic use of RFA with other modalities, such as photodynamic therapy, is also promising (86). Furthermore, deep integration of artificial intelligence and precision medicine will reshape the diagnostic–therapeutic paradigm. Deep-learning algorithms can be used to analyze EUS and clinical data to identify subtle high-risk features, to auto-segment lesions, and to predict complications or treatment response (87, 88). Combined with NGS of cyst fluid, the goal is to generate a personalized, optimal treatment plan for each PCL patient (89). Despite demonstrating significant potential and accumulating encouraging clinical evidence in the treatment of PCLs, the path for EUS-RFA to become a standardized, routine treatment option still faces challenges and limitations, which also delineate the direction for future research.

## 7 Summary and clinical outlook

EUS-RFA has established itself as a viable, minimally invasive therapeutic modality for managing selected PCLs. It effectively bridges a critical management gap for patients with lesions possessing malignant potential who are suboptimal surgical candidates due to comorbidities or personal preference. Current evidence robustly supports its favorable safety profile and high technical efficacy, particularly in the ablation of mural nodules and the epithelium of mucinous cysts, with the primary goal of reducing the risk of malignant progression.

The successful integration of EUS-RFA into clinical practice hinges on stringent, multidisciplinary team-based patient selection, utilizing the criteria outlined in this review. Optimal outcomes are achieved in specialized centers with consolidated expertise, adhering to standardized procedural protocols. However, the application of this technology must be contextualized within the limitations of the present evidence base, which predominantly consists of non-randomized, observational studies. Long-term data on oncological outcomes exceeding 5 years and direct comparative effectiveness versus surgical resection are still maturing.

Therefore, EUS-RFA should not be viewed as a replacement for surgery in standard-risk patients but as a complementary and valuable alternative within a personalized treatment strategy. Its adoption should be guided by careful risk–benefit analysis and shared decision-making. Future efforts must focus on standardizing procedural protocols, validating robust response assessment criteria, and obtaining high-level comparative evidence through randomized trials. As these data accumulate, EUS-RFA is poised to play an increasingly defined and significant role in the precision management of pancreatic cystic neoplasms.
